# Fecal microbiota in horses with asthma

**DOI:** 10.1111/jvim.15748

**Published:** 2020-03-04

**Authors:** Mathilde Leclere, Marcio C. Costa

**Affiliations:** ^1^ Clinical Sciences Department Université de Montréal Québec Canada; ^2^ Veterinary Department of Biomedical Sciences Université de Montréal Québec Canada

**Keywords:** gut‐lung axis, heaves, horse, microbiome, recurrent airway obstruction

## Abstract

**Background:**

Gastrointestinal microbiota can be influenced by several factors, including diet and systemic inflammation, and in turn could act as a modulator of the allergic response. Fecal microbiota of horses with asthma has not been described.

**Hypothesis/Objectives:**

Analyze the bacterial fecal microbiota of horses with and without asthma under different environment and diet conditions, during both remission and exacerbation.

**Methods:**

Prospective observational study. Feces from 6 asthmatic and 6 healthy horses were collected under 3 different conditions: on pasture, housed indoors receiving good quality hay (“good hay”), and housed indoors receiving poor quality hay (“dusty hay”). Sequencing was performed using an Illumina MiSeq platform and data were processed using the software mothur v.1.41.3 and LEfSe.

**Results:**

In horses with asthma, low‐abundance bacteria were more affected by changes in environment and diet (ie, when horses were experiencing an exacerbation), as shown by changes in membership and results from the LEfSe analysis. There was a significant increase in the relative abundance of Fibrobacter in healthy horses eating hay, a change that was not observed in horses with asthma.

**Conclusions and Clinical Importance:**

The intestinal microbiota of horses with asthma does not adapt in the same way to changes in diet and environment compared to the microbiota of healthy horses. Mechanisms explaining how airway obstruction and inflammation could influence the intestinal microbiota and how in turn this microbiota could modulate systemic inflammation in asthmatic horses deserves further investigation.

**Abbreviations**
AMOVA: analysis of molecular variance
ANOVA: analysis of variance
CBC: complete blood count
DNA: deoxyribonucleic *acid*
LDA: linear discriminant analysis
LEfSe: linear discriminant analysis effective size
OTU: operational taxonomic units
PCoA: principal coordinate analysis
RDP: Ribosomal Database Project
rRNA: *ribosomal* ribonucleic *acid*

## 1 INTRODUCTION

Asthma in horses is characterized by reversible bronchospasm and airway inflammation upon exposure to inhaled antigens. It is a complex disease that includes individual susceptibility to allergen exposure, variable levels of severity, and different inflammatory phenotypes.^1^, ^2^, ^3^ Exacerbations are characterized by the presence of clinical signs and are mainly triggered by exposure to aerosolized allergens found in hay and bedding, including fungal particles, mites, and endotoxin.^3^ The role of these environmental inhaled allergens is well documented, but questions remain about factors contributing to the development of asthma in horses, as well as why some horses affected at a young age with mild asthma do not develop the severe form (formerly known as “heaves”) and why the severity of exacerbations can vary over time. The role of gastrointestinal microbiota as a potential modulator of the allergic response has not been investigated in horses with asthma.

Normal gastrointestinal microbiota seems essential to the development of tolerance to antigens. This is not limited to the bacteria and other microorganisms themselves, but also to the numerous mediators produced by these organisms, such as peptidoglycans, endotoxin, and short‐chain fatty acids.^4^ Our knowledge of the role of the intestinal microbiota in modulating allergic responses is in part based on the study of germ‐free mice. Such mice are more susceptible to certain types of allergic reactions^4^ and develop a T‐helper 2‐mediated response when colonized with microbiota of little diversity, leading to a hyper‐reactive immune response to commensal bacteria.^5^ There also is strong evidence that dysbiosis in infancy influences the risk of developing asthma and other allergic diseases.^6^ Beyond the development of tolerance at a young age, change in the gastrointestinal microbiota in older animals could contribute to systemic and pulmonary inflammation by changes in these metabolites in the gastrointestinal tract. In humans, the fecal microbiota of adults with asthma was shown to differ from that of healthy controls, and an association has been observed among fecal microbiota, lung function, and allergen sensitization.^7^ It is too early however to speculate on a causative relationship, because it also could be that the systemic inflammation observed in humans and horses with asthma^8^, ^9^, ^10^ can induce changes in the gastrointestinal microbiota. Our goal therefore was to study the intestinal microbiota of horses with severe asthma when they are in remission (housed on pasture) and twice during exacerbation (housed in a barn and fed good quality hay and dusty hay) compared to healthy horses housed in the same environments. We hypothesized that the intestinal microbiota would be influenced by diet and housing conditions (“environment”), as well as disease status when horses with asthma are experiencing an exacerbation.

## 2 METHODS

### 2.1 Animals and study design

Fecal samples were collected from a previously described cohort of adult horses with asthma and age‐matched controls.^11^ Six horses (2 geldings, 4 mares) with a history of reversible episodes of airway obstruction and 6 healthy controls (6 mares) housed in the same barn were enrolled (n = 12). Control horses were considered healthy based on history, physical examination and complete blood count (CBC). No antimicrobials or corticosteroids were administered for at least 3 months and 1 month, respectively, before the beginning of the study and none had a history of gastrointestinal disease in the previous 6 months. Horses were housed together on grass pasture, in a large field with no other horses, with no additional grain for at least 3 weeks before fresh fecal samples were collected from the rectum of each horse (“pasture”). They then were housed indoors in individual stalls and fed good‐quality hay (“good hay”) and then poor‐quality hay harvested wet the same year to increase mold growth (“dusty hay”) in a cross‐over design for 3 weeks each. Nutritional analysis of the grass and hay were not performed. Hay was fed in the morning and in the late afternoon, and they did not receive grain but could receive occasional treats (carrots). All horses were turned out together in a dry paddock for 2 to 4 hours daily. Experimental procedures were performed in accordance with the Canadian Council for Animal Care guidelines and were approved by the Animal Care Committee of the Université de Montréal (# 15Rech1760).

### 2.2 Samples collection and DNA extraction

Fecal samples were put on ice until they were frozen at −80°C, within 2 hours of collection. Total DNA was extracted using the DNeasy PowerSoil Kit (Qiagen Toronto, ON, CA) following the manufacturer's instructions.

### 2.3 High throughput sequencing

The V4 region of the bacterial 16S rRNA gene was amplified by PCR using the following primers: 515F (GTGCCAGCMGCCGCGGTAA) and 806R (GGACTACHVGGGTWTCTAAT) as previously recommended.^12^ Sequencing was performed using an Illumina MiSeq platform, using the V2 reagent kit (2 × 250 cycles) at the Genome Quebec Innovation Centre. Sequences are available at the NCBI Sequence Read Archive (access number PRJNA608016).

### 2.4 Data analysis

Sequence data were processed using the software mothur v.1.41.3,^13^ following the Standard Operating Procedure previously described.^14^ Good quality reads were clustered in operation taxonomic units (OTU) at the species, genus, and family level (> 97, 94, and 92% similarity, respectively) and classified according to the Ribosomal Database Project (RDP) databank. Sequences that were present ≤5 times were removed from analysis. The Chao richness estimator, Simpson index, and Shannon index were used for characterization of alpha diversity at the family, genus, and species levels of taxonomy. Beta diversity evaluating similarities among samples was addressed by the Jaccard index and the Yue and Clayton index to compare, respectively, community membership (that considers the different taxa) and structure (that considers the different taxa and their distribution within the community). Beta diversity was explored visually using principal coordinate analysis (PCoA). Finally, the get.community type command was used to cluster microbial communities into enterotypes.

### 2.5 Statistical analysis

Normality was assessed by visual inspection of the data and use of the Kolmogorov‐Smirnov test. Data were log‐transformed when indicated. Indices of alpha diversity and relative abundance of the 7 most common phyla and 12 most common genera were analyzed using a repeated measures 2‐way ANOVA on log‐transformed data, considering group (healthy or asthmatic) and environment as variables. When significant effect was present, post‐hoc comparisons were performed between groups and comparing hay with pasture, using the Fisher LSD test, without correcting for multiple comparisons. Unless mentioned otherwise, ± SD is reported. A *P* value of .05 was used to assign significance. Beta diversity (community membership and structure) were compared using the Parsimony (*t* test) and the analysis of molecular variance (AMOVA) tests.

Differences between groups and environments were further explored using linear discriminant analysis effective size (LEfSe, version 1.0),^15^ which uses factorial Kruskal‐Wallis sum‐rank and a subsequent pairwise test (Wilcoxon rank‐sum) to detect features with biological significance by comparing the abundance in all populations, including those with low abundance. As a last step, LEfSe uses linear discriminant analysis (LDA) to estimate the effect size of each differentially abundant feature. Alpha values for the factorial Kruskal‐Wallis test among classes and for the pairwise Wilcoxon test between subclasses were set to .05. Threshold on the logarithmic LDA score for discriminative features was set to 2.0 and the strategy for multiclass analysis was set to all‐against‐all (stricter).

## 3 RESULTS

### 3.1 Horses

Weights, ages, lung function, and bronchoalveolar lavage cytology were previously reported.^11^ Briefly, there were no differences between groups for age and sex, and as expected, and only horses with asthma developed significant airway obstruction (measured by impulse oscillometry) and severe airway inflammation (bronchoalveolar lavage neutrophilia) with hay feeding and indoor environment.

### 3.2 Number of reads/metrics

A total of 1 371 320 reads passed all quality filters and were retained for microbiota analysis from a total of 3 287 914 reads. A subsample of 39 344 reads per sample was used for alpha diversity analysis to decrease potential bias caused by nonuniform sample sizes. Good's coverage after subsampling and removing sequences found ≤5 times was on average 97.50 ± .35%.

### 3.3 Relative abundance of the most common phyla and genera

**Figure 1:**
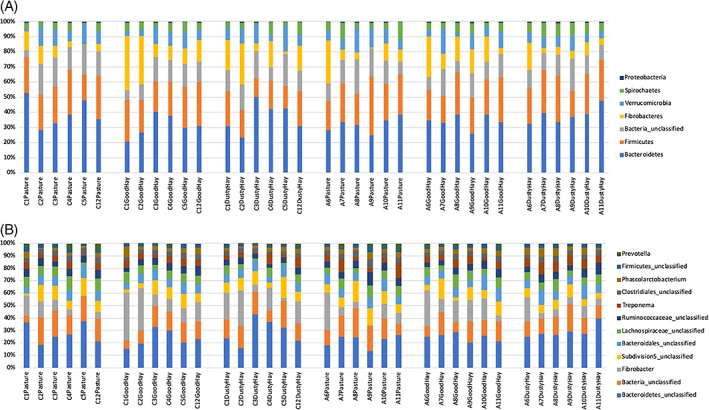
Relative abundance of predominant bacteria at the phylum (A) and genus (B) levels. Six horses with and without asthma (samples starting with A and C, respectively) were housed on pasture (“Pasture”), indoors receiving good quality hay (“GoodHay”), and indoors receiving poor quality hay (“DustyHay”). Only the 7 most common phyla and 12 most common genera are represented

**Figure 2:**
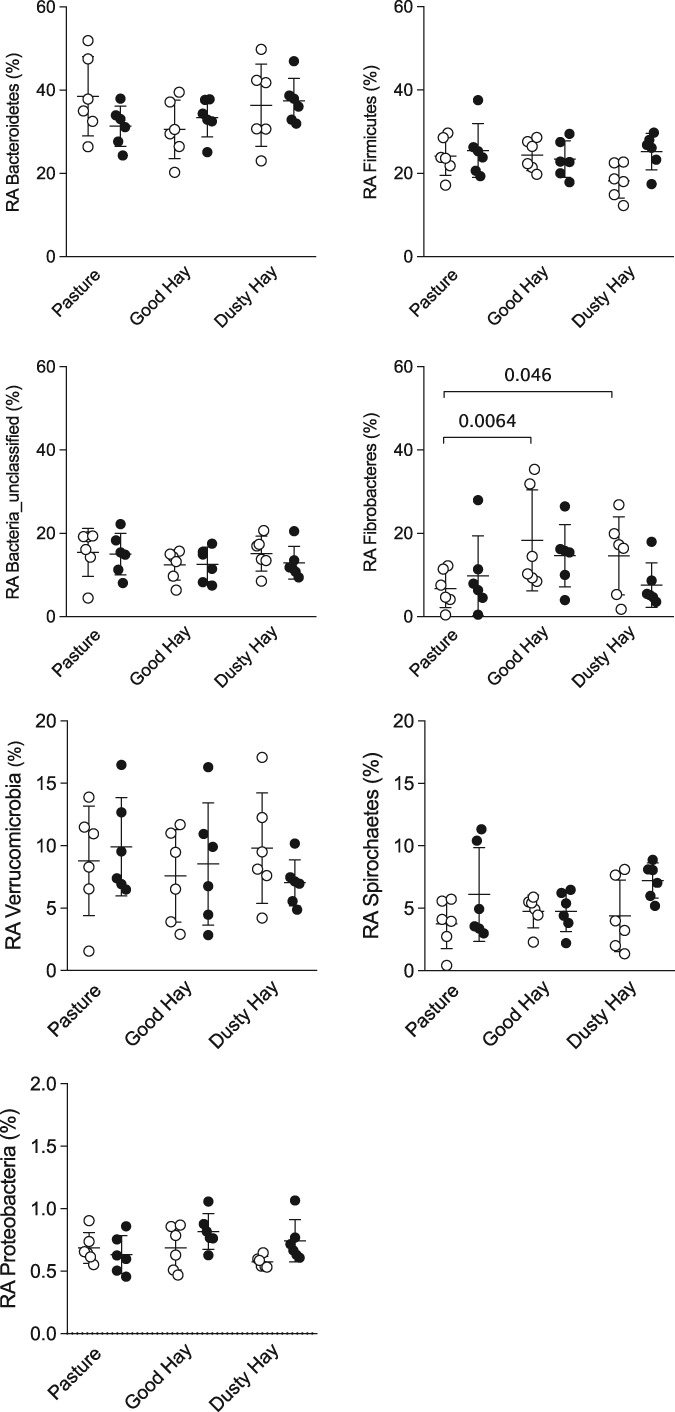
Relative abundance (RA) of the seven most common phyla by group and environment. Open circles: 6 healthy horses. Black circles: 6 horses with asthma. Bars represent mean and SD. For Fibrobacteres, there was an overall significant effect of the environment (*P* < .01), and a significant increase when horses were fed hay compared to pasture. Repeated measure ANOVA were done on log‐transformed data, followed by uncorrected Fisher's LSD

The relative abundances of the most common phyla and genera found in each group in the different environments are presented in Figure 1. Sequences were classified into 19 different phyla, 7 of them accounting for >98% of the total number of sequences. The majority of OTUs were assigned to the Bacteroidetes phylum (34% on average), the Firmicutes phylum (24%), followed by bacteria that were unclassified at the phylum level (14%). Among the 7 most common phyla, no significant differences were found between groups and environments, except for Fibrobacteres (bacteria involved in cellulose degradation) with an overall significant effect of the environment (*P* < .01; 2‐way ANOVA on log‐transformed data). Post‐hoc tests (uncorrected Fisher's LSD) showed that the increase of these cellulolytic bacteria with the hay/barn environment was only present in healthy horses, not in asthmatic horses (Figure 2). Also, a significant horse effect was found for Fibrobacteres (*P* = .01).

**Figure 3:**
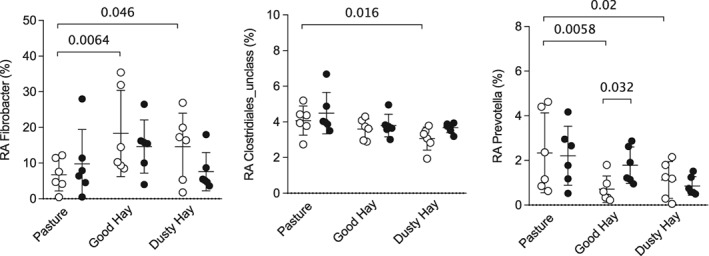
Relative abundance (RA) of three genera affected by environmental and diet conditions by group and environment. Open circles: 6 healthy horses. Black circles: 6 horses with asthma. Bars represent mean and SD. There was an overall significant effect of the environment and diet for these 3 genera (*P* < .01, .02, and < .01 for Fibrobacter, unclassified Clostridiales, and Prevotella, respectively). Repeated measure ANOVA were done on log‐transformed data, followed by uncorrected Fisher's LSD

The 12 most common genera (> 1% abundance) found in the samples represented together 91.6% of all sequences, and 93.11% of the 7 most common phyla. The 2 most common genera were unclassified Bacteroidetes and unclassified bacteria (representing overall 23.55% and 13.93% of all sequences, respectively). Fibrobacter was the third most abundant genus and, as expected because this genus comprised nearly all reads of the Fibrobacteres phylum in our samples, a significant increase after hay feeding in healthy horses also was observed (*P* < .01; 2‐way ANOVA on log‐transformed data). In addition, change in environment was associated with a decrease of unclassified Clostridiales (*P* = .02) and Prevotella, (*P* < .01), with post‐hoc tests also significant only in the control group (Figure 3). Also, significant horse effects were found for Fibrobacter, the unclassified Bacteroidales, and Prevotella (all *P* values < .05).

### 3.4 Relative abundance analyzed using the LEfSe method at the genus level

**Figure 4:**
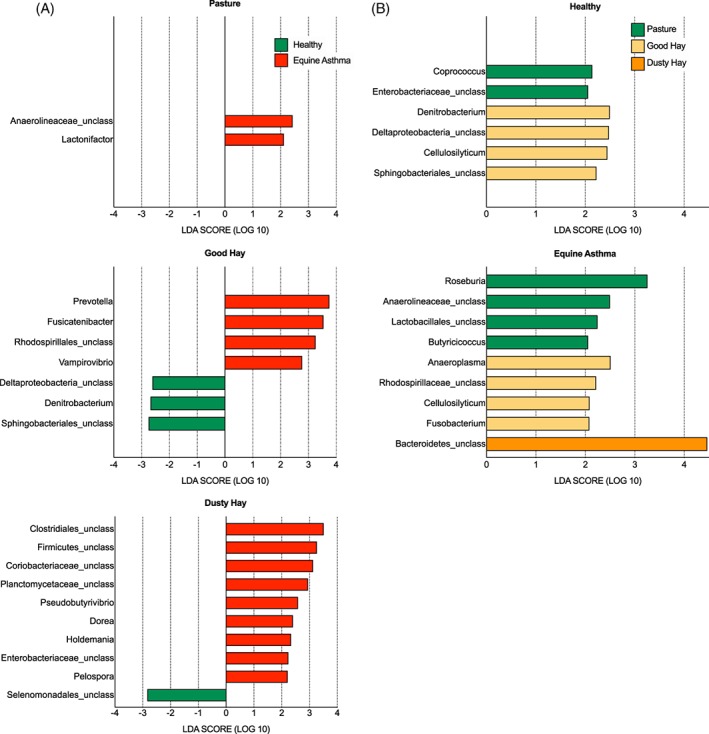
Linear discriminant analysis effect size (LEfSe) analysis. A, LEfSe analysis showing genera that were significantly differentially abundant between groups (healthy in green, equine asthma in red). B, Environments (pasture in green, good hay in yellow, dusty hay in orange) (all LDA scores >2). LDA, linear discriminant analysis; Unclass, unclassified at the genus level

The difference in relative abundance at the genus level also was analyzed using LEfSe. This method combines statistical significance with biological consistency and effect size estimation, and allows for comparison of low‐abundance genera. When horses were in remission on pasture, only 2 genera were significantly overrepresented in the asthma group (Figure 4A), whereas there were 4 and 9 genera associated with horses experiencing exacerbation and receiving good and dusty hay, respectively. At the same time, 3 and 1 genera were overrepresented in controls receiving good and dusty hay, respectively. The LefSe analysis confirmed the differences between groups in relative abundances of Prevotella under the good hay condition, and identified other genera that were overrepresented in horses with asthma under dusty hay conditions, including unclassified Clostridiales and Firmicutes (genera with *P* values of .08 and .11 between groups, respectively, with post‐hoc tests in the ANOVA analysis of relative abundance). Overall, this finding suggests that there are more differences between groups when horses are experiencing exacerbation, despite eating the same diet, possibly because of their disease status.

Out of the 15 genera overrepresented in horses with asthma, 8 belonged to the Firmicutes phylum (5 of them to the Clostridia class including 3 Lachnospiraceae), but only 1 genus of this phylum, an unclassified Selenomonadales, was associated with healthy horses. Healthy horses had 2 genera overrepresented on pasture and 4 on good hay, but none were significant on dusty hay, and the expected changes in Fibrobacter, Prevotella, or the unclassified Clostridiales (observed in healthy horses, Figure 3A) were not significant with this analysis (Figure 4B).

Horses with asthma had more changes associated with their diet and exacerbation status. The unclassified Bacteroidetes was the genus with the highest LDA score and was only overrepresented when horses with asthma were in the dusty hay conditions. With the ANOVA, there was a nonsignificant effect of environment (*P* = .08) with post‐hoc test *P* value of .03 for asthmatic horses between pasture and dusty hay conditions. Interestingly, both groups had increased Cellulosilyticum (Lachnospiraceae, Firmicutes) with good hay, another cellulose‐degrading bacteria.

### 3.5 Alpha diversity: richness and diversity within samples

**Figure 5:**
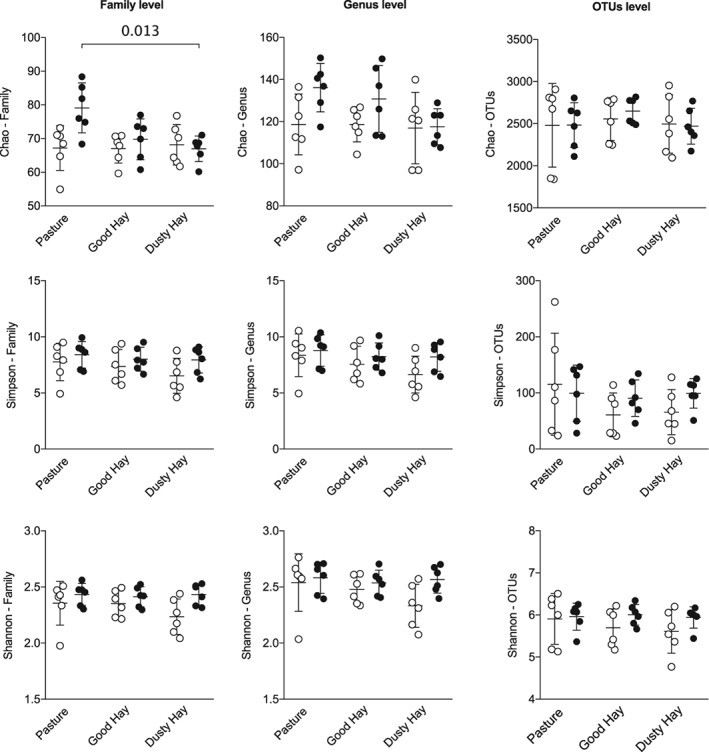
Indices of alpha diversity at the family, genus, and species level of taxonomy. Open circles: 6 healthy horses. Black circles: 6 horses with asthma. Bars represent mean and SD. At the family level, there was an overall significant interaction effect for the Chao index (*P* = .02, 2‐way ANOVA on log‐transformed data), with a significant decrease of richness between pasture and dusty hay (*P* = .01, uncorrected Fisher LSD) and a significant group effect for the Shannon index (*P* = .02). Fisher's LSD did not show significant pair‐wise comparisons and the differences observed at the family level were not found at the genus or species levels

The Chao richness estimator, the Simpson index, and the Shannon diversity index were used for characterization of alpha diversity at the family, genus, and species levels of taxonomy (Figure 5). Overall, few significant changes were found between groups or environment in terms of richness and diversity. There was only a significant interaction between groups and environments for the Chao index (richness) at the family level (*P* = .02, 2‐way ANOVA on log‐transformed data), with a significant decrease of richness between pasture and dusty hay for asthmatic horses only (*P* = .01, uncorrected Fisher LSD). This decrease was because of higher baseline richness on pasture, not lower richness when horses were symptomatic. Still at the family level, the Shannon index (more influenced by rare taxa than the Simpson index) but not the Simpson index was significantly higher in horses with asthma (*P* value for group effect .02 and .05, respectively). Fisher's LSD did not show significant pair‐wise comparisons, and the differences observed at the family level were not found at the genus or species levels.

### 3.6 Beta diversity: PCoA plots and AMOVA tests at the genus level

**Figure 6:**
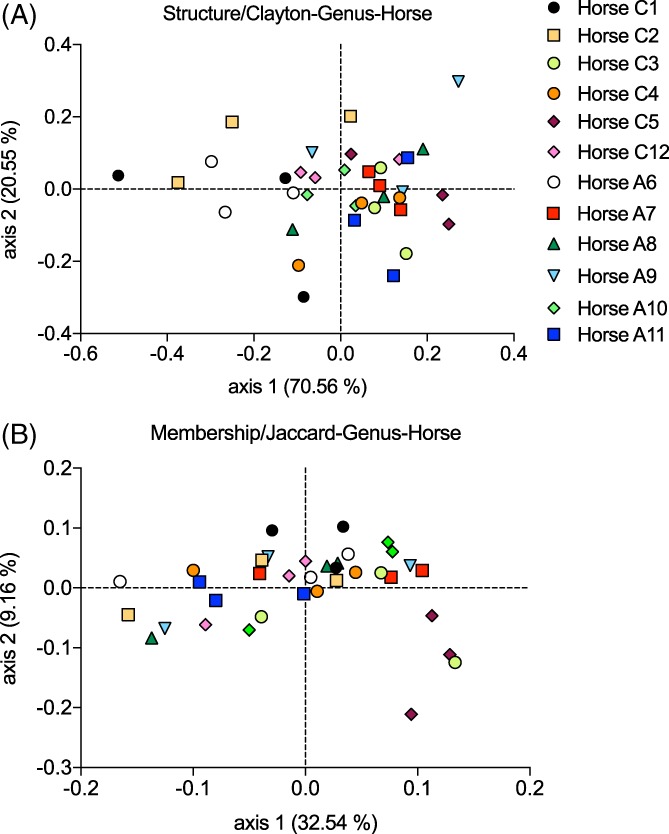
Principal coordinate analysis (PCoA) of bacterial communities at the genus level by horses. Bidimentional representation of the principal coordinate analysis of bacterial communities’ structure using the Yue and Clayton analysis (A) and membership using the Classic Jaccard analysis (B). Samples from these 6 healthy horses and 6 asthmatic horses in 3 different environments (pasture, good quality hay, and dusty hay) significantly cluster together. Dots with the same shape and color clustered significantly together (AMOVA, *P* = .002 for structure (A) and *P* < .001 for membership (B))

**Figure 7:**
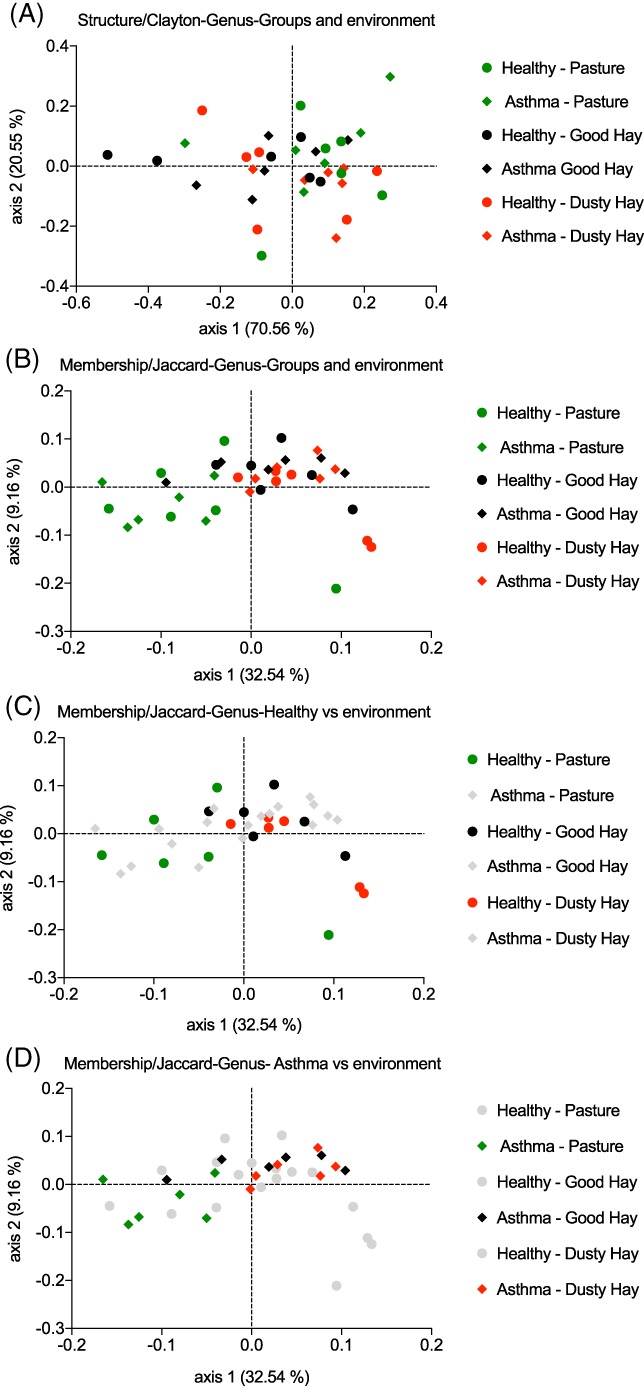
Principal coordinate analysis (PCoA) of bacterial communities at the genus level by groups and environments. Bidimentional representation of the principal coordinate analysis of bacterial communities’ structure using the Yue and Clayton analysis (A) and membership using the Classic Jaccard analysis (B, C, and D). Samples from these 6 healthy horses and 6 asthmatic horses in 3 different environments (green: pasture, black: good quality had, red: dusty hay). C and D, The groups removed from analysis were greyed out. A, The samples’ structure did not cluster significantly by group or by environment. B, The samples’ membership did not cluster by groups when environments were analyzed separately (circles versus diamonds within the same color) but clustered by environment (pasture versus good hay or dusty hay, AMOVA, *P* < .001). This clustering was not significant when only healthy horses were analyzed (C) but was significant in asthmatic horses (D) (pasture versus good hay or dusty hay, AMOVA, *P* ≤ .001)

Beta diversity (structure and membership) was compared between groups with the AMOVA test at the genus level. In terms of structure (genera and their relative abundances), the samples clustered significantly by horse (AMOVA, 12 horses, *P* = .002; Figure 6A), but not by group (all samples taken into account, and in each environment to avoid repeated contribution of the same horse more than once; *P* value > .13 for all) nor was there a significant environment effect. The closest to a significant effect of the environment was when all horses were analyzed together in the 3 environments (AMOVA 3 environments, *P* = .07) or when pasture was compared to dusty hay (AMOVA 2 environments, *P* = .07; Figure 7A).

In terms of membership (genera present or absent, not taking in account their abundances), the samples also clustered significantly by horse (AMOVA, 12 horses, *P* < .001; Figure 6B). A significant group effect was found when all horses were analyzed together (ie, with each horse contributing more than once; AMOVA *P* = .03) but no significant difference was observed when environments were analyzed separately (*P* = .08 on dusty hay, *P* > .19 in the other environments; Figure 7B). A strong effect of environment was observed with significant differences between hay (good or dusty) versus pasture (AMOVA, *P* < .001), but not between the types of hay (*P* = .87; Figure 7B). When each group was analyzed separately, this effect still was present between pasture and good hay as well as pasture and dusty hay for asthmatic horses (AMOVA, *P* ≤ .001 for pair‐wise comparisons; Figure 7D). In healthy horses, no significant effect was found between pasture and good hay (AMOVA, *P* = .21) nor between pasture and dusty hay (AMOVA, *P* = .2; Figure 7C). Neither group had significant differences between types of hay.

### 3.7 Enterotypes

**Figure 8:**
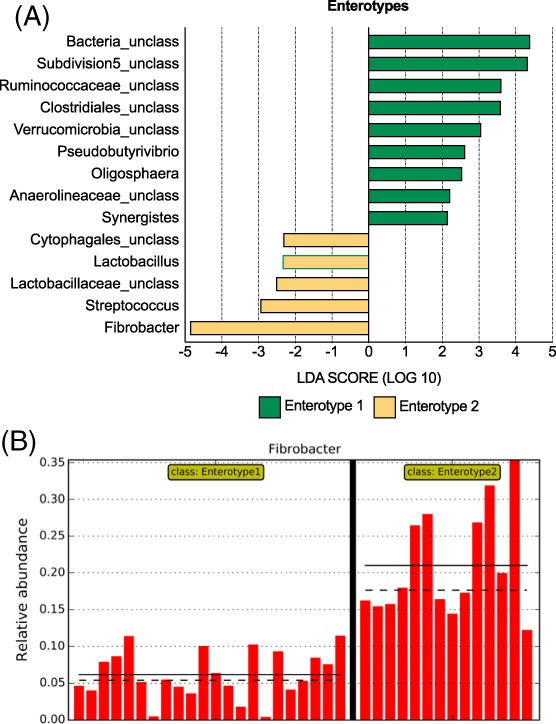
Linear discriminant analysis effect size (LEfSe) analysis. A, LEfSe analysis showing genera that were significantly differentially abundant between enterotype 1 (“pasture enterotype”) and enterotype 2 (“hay enterotype”). All LDA scores >2. B, Relative abundance of fibrobacter, the major discriminant between the two enterotypes. Straight lines represent the mean, dotted lines represent the median. LDA, linear discriminant analysis; Unclass, unclassified at the genus level

The lower Laplace number indicated that the communities best fitted into 2 distinct enterotypes. All but 2 horses on pasture were associated with enterotype 1 (“Pasture enterotype”) and 12 of 14 samples associated with enterotype 2 were from horses eating hay (“Hay enterotype”; *P* = .08, Fisher's exact test). The LEfSe analysis was used to determine which genera were associated with each enterotype, and the major discriminant was higher abundance of Fibrobacter among hay‐fed horses (Figure 8).

## 4 DISCUSSION

### 4.1 Summary

Our study confirms that diet and environment have significant effects on fecal microbiota of horses and that a change of environment of 3 weeks is enough time to induce significant changes in the intestinal microbiota of healthy and asthmatic animals. More importantly, it also shows that the intestinal microbiota of horses with and without asthma housed together does not adapt in the same way to environmental changes. Although a change from grass pasture to a hay diet is associated in healthy horses with an increase in Fibrobacter and a decrease in Prevotella and unclassified Clostridiales, the low‐abundance genera appear to be more affected in horses with asthma, resulting in a significant change in beta diversity (membership) and more differentially abundant communities observed with the LEfSe analysis. Thus, changes observed in controls are more centered on bacteria largely represented (>1% abundance), and these core constituents of the microbiota do not adapt as well to change in environment in asthmatic horses. In asthmatic horses, change in diet and environment affects the relative composition of low‐abundance taxa, which could explain changes in alpha diversity indices that are more affected by rare taxa (Shannon index but not Simpson index increased in asthmatic horses, Jaccard membership but not Yue and Clayton structure, LEfSe analysis but not in the relative abundances of the most common phyla or genera). Although our study is merely descriptive, it could be speculated that systemic inflammation induced by the upregulated immune system of asthmatic horses might influence the intestinal environment.^16^ In return, altered intestinal microbiota could affect systemic and pulmonary inflammation by changes in the absorption of short‐chain fatty acids or bacterial (ie “bacterial wall”)‐wall components (eg, endotoxin) and local or distant cytokine upregulation.^4^, ^17^

### 4.2 Differences between healthy and asthmatic horses

In a previous study, we showed that pulmonary, oral, and nasal microbiota are influenced by environmental conditions, but only the microbiota of the lungs differs between horses with and without asthma.^11^ The difference was mainly present when horses with asthma were experiencing exacerbation. Here, we demonstrated that the intestinal microbiota also differs between healthy and asthmatic horses, again mainly when horses with asthma are experiencing exacerbation (ie, exposed to hay and housed indoors). In horses with asthma, exposure to indoors and hay dust induces clinical exacerbation characterized by increases in airway resistance, pulmonary and systemic inflammation, and with decreased oxygenation,^9^, ^11^, ^18^ factors that could affect gastrointestinal microenvironment and motility. Excess airway mucus that is coughed up and swallowed also could affect the gastrointestinal tract microbiota by changing the relative proportion of airway and oral bacteria swallowed. Fewer differences also are observed when horses are on pasture and asymptomatic, suggesting that changes in fecal microbiota could be secondary to exacerbation. On the other hand, it is possible that the gut microbiota is altered even in horses in remission (differences not observed here possibly because of the low number of animals) and that chronic dysbiosis prevents normal adaptation to the new diet and environmental conditions. A third hypothesis is that horses with chronic asthma could have been less exposed to hay over the years, as a strategy to control their condition, changing their capacity to quickly adapt to a hay diet. Taken together, these factors highlight the need for these observations to be replicated by independent researchers in more controlled conditions.

Among the differences observed, some are reminiscent of changes observed in adult humans with asthma, such as increased Firmicutes relative to Bacteroidetes, decreased Lachnospiraceae family, and overrepresentation of Prevotella in a subset of patients with poor lung function.^7^ Decreased short‐chain fatty acids from feces of adult asthmatics experiencing exacerbation (total and acetate, butyrate and proprionate) compared to healthy humans also is described,^19^ reflecting a change in the gastrointestinal microbiota of asthmatics. In mice, an increase in dietary fiber alters the gut microbiota, increases circulating short‐chain fatty acids, decreases the relative abundance of Firmicutes, and decreases susceptibility to allergic lung inflammation.^20^ In our study, horses with asthma lacked the expected increase in Fibrobacter and had overrepresentation of Firmicutes after dusty hay feeding, similarly to the changes observed in mice with more inflammation and a low fiber diet. Short‐chain fatty acids were not measured in our study and, at this stage, causative relationship remains speculative. It is possible, however, that the difficulty encountered in reproducing not only inflammation but also airway obstruction and clinical signs with inhalation challenges are limited by the fact that they bypass the gastrointestinal‐lung axis.^21^, ^22^ Finally, unlike a recent study that showed decreased diversity in adults with asthma,^23^ we did not observe differences in diversity. Differences could arise from the fact that most asthmatic patients are on corticosteroids because medication is not typically withheld for such studies.

### 4.3 Differences between environments

Environmental change (diet and housing) induced strong changes in the fecal microbiota of studied horses, as shown by changes in community membership (presence/absence of taxa) in horses with asthma, and changes in relative abundance of common genera in healthy horses. These findings are not surprising but confirm that it takes ≤3 weeks to observe significant changes in the intestinal microbiota of horses. These shifts with diet and seasons are considered normal in healthy horses, including the increased relative abundance of Fibrobacters with introduction of haylage to the diet of horses on pasture.^24^ Other studies showed that bacterial communities changed within 4 days of transitioning horses from an ensiled alfalfa grass and corn grain diet to pasture,^25^ and within 2 to 4 days when humans transition from a plant‐based to an animal‐based diet or vice versa.^26^

It was also interesting that different hay quality did not significantly affect the fecal microbiota of studied horses. Indeed, samples clustered into 2 major groups (enterotypes), and these were mainly separated by the marked overrepresentation of Fibrobacter in enterotype 2, found mainly in horses receiving hay.

### 4.4 Intestinal microbiota of horses in other studies

The 7 most common phyla found in our study were the same as in a previous study.^27^ The most notable difference is that Bacteroidetes accounted for >34% of sequences in our study, but <5% in the previous study,^27^ which is likely because of the use of different primers.^12^ Fibrobacteres, increased in our study in healthy horses eating hay, was found in higher abundance after weaning.^28^ It also is described as part of the core microbiota (ie, not necessarily the most abundant taxa, but bacteria found in most horses) of the hind gut of horses,^29^ but tends to be low in relative abundance in horses on pasture or eating silage (<1%),^25^ and even lower in other studies, even in horses eating hay.^30^

### 4.5 Limitations

The main limitation of our study is the small sample size in each group, which may have affected the ability to identify certain differences. Also, the relatively large number of unclassified bacteria at every level of taxonomy makes inference to potential function of the identified communities limited. It also was not possible to impute causality to our observations, especially because exacerbation of asthma in horses is intrinsically linked to a change in diet. This could change in the future, by inducing exacerbation without changing the diet with methods using hay mixed with oil to maintain horses in remission thus decreasing inhaled antigens without changing the type of diet.^31^

## CONFLICT OF INTEREST DECLARATION

Authors declare no conflict of interest.

## OFF‐LABEL ANTIMICROBIAL DECLARATION

Authors declare no off‐label use of antimicrobials.

## INSTITUTIONAL ANIMAL CARE AND USE COMMITTEE (IACUC) OR OTHER APPROVAL DECLARATION

All animal manipulations were performed in accordance with the guidelines of the Canadian Council for Animal Care, and the protocol was approved by the Animal Care and Use Committee of the University of Montreal (approval number: 15Rech1760).

## HUMAN ETHICS APPROVAL DECLARATION

Authors declare human ethics approval was not needed for this study.

## References

[1] Gerber V , Tessier C , Marti E . Genetics of upper and lower airway diseases in the horse. Equine Vet J. 2015;4:390‐397. 10.1111/evj.12289 24773614

[2] Bond S , Leguillette R , Richard EA , et al. Equine asthma: integrative biologic relevance of a recently proposed nomenclature. J Vet Intern Med. 2018;32:2088‐2098. 30294851 10.1111/jvim.15302 PMC6271326

[3] Couetil LL , Cardwell JM , Gerber V , et al. Inflammatory airway disease of horses‐revised consensus statement. J Vet Intern Med. 2016;30:503‐515. 26806374 10.1111/jvim.13824 PMC4913592

[4] Frati F , Salvatori C , Incorvaia C , et al. The role of the microbiome in asthma: the gut−lung axis. Int J Mol Sci. 2018;20:E123. 30598019 10.3390/ijms20010123 PMC6337651

[5] Gensollen T , Blumberg RS . Correlation between early‐life regulation of the immune system by microbiota and allergy development. J Allergy Clin Immunol. 2017;139:1084‐1091. 28390575 10.1016/j.jaci.2017.02.011 PMC5402752

[6] Martinez FD , Guerra S . Early origins of asthma: role of microbial dysbiosis and metabolic dysfunction. Am J Respir Crit Care Med. 2018;197:573‐579. 29048927 10.1164/rccm.201706-1091PP PMC6005239

[7] Begley L , Madapoosi S , Opron K , et al. Gut microbiota relationships to lung function and adult asthma phenotype: a pilot study. BMJ Open Respir Res. 2018;5:e000324. 10.1136/bmjresp-2018-000324 PMC6157510 30271607

[8] Leclere M , Bedard C , Cortes‐Dubly ML , Lavoie JP . Blood hypercoagulability and systemic inflammation in horses with heaves. Vet J. 2015;206:105‐107. 26164529 10.1016/j.tvjl.2015.04.012

[9] Lavoie‐Lamoureux A , Leclere M , Lemos K , Wagner B , Lavoie JP . Markers of systemic inflammation in horses with heaves. J Vet Intern Med. 2012;26:1419‐1426. 22925172 10.1111/j.1939-1676.2012.00993.x

[10] Bjermer L . Time for a paradigm shift in asthma treatment: from relieving bronchospasm to controlling systemic inflammation. J Allergy Clin Immunol. 2007;120:1269‐1275. 18073122 10.1016/j.jaci.2007.09.017

[11] Fillion‐Bertrand G , Dickson RP , Boivin R , Lavoie JP , Huffnagle GB , Leclere M . Lung microbiome is influenced by the environment and asthmatic status in an equine model of asthma. Am J Respir Cell Mol Biol. 2019;60:189‐197. 30183323 10.1165/rcmb.2017-0228OC

[12] Walters W , Hyde ER , Berg‐Lyons D , et al. Improved bacterial 16S rRNA gene (V4 and V4‐5) and fungal internal transcribed spacer marker gene primers for microbial community surveys. mSystems. 2015;1:e00009‐15. 10.1128/mSystems.00009-15 PMC5069754 27822518

[13] Schloss PD , Westcott SL , Ryabin T , et al. Introducing mothur: open‐source, platform‐independent, community‐supported software for describing and comparing microbial communities. Appl Environ Microbiol. 2009;75:7537‐7541. 19801464 10.1128/AEM.01541-09 PMC2786419

[14] Kozich JJ , Westcott SL , Baxter NT , Highlander SK , Schloss PD . Development of a dual‐index sequencing strategy and curation pipeline for analyzing amplicon sequence data on the MiSeq Illumina sequencing platform. Appl Environ Microbiol. 2013;79:5112‐5120. 23793624 10.1128/AEM.01043-13 PMC3753973

[15] Segata N , Izard J , Waldron L , et al. Metagenomic biomarker discovery and explanation. Genome Biol. 2011;12:R60. 21702898 10.1186/gb-2011-12-6-r60 PMC3218848

[16] Yitbarek A , Taha‐Abdelaziz K , Hodgins DC , et al. Gut microbiota‐mediated protection against influenza virus subtype H9N2 in chickens is associated with modulation of the innate responses. Sci Rep. 2018;8:13189. 30181578 10.1038/s41598-018-31613-0 PMC6123399

[17] Mirkovic B , Murray MA , Lavelle GM , et al. The role of short‐chain fatty acids, produced by anaerobic bacteria, in the cystic fibrosis airway. Am J Respir Crit Care Med. 2015;192:1314‐1324. 26266556 10.1164/rccm.201505-0943OC PMC4731701

[18] de Lagarde M , Rodrigues N , Chevigny M , Beauchamp G , Albrecht B , Lavoie JP . N‐butylscopolammonium bromide causes fewer side effects than atropine when assessing bronchoconstriction reversibility in horses with heaves. Equine Vet J. 2014;46:474‐478. 24423012 10.1111/evj.12229

[19] Ivashkin V , Zolnikova O , Potskherashvili N , et al. Metabolic activity of intestinal microflora in patients with bronchial asthma. Clin Pract. 2019;9:1126. 30931087 10.4081/cp.2019.1126 PMC6401556

[20] Trompette A , Gollwitzer ES , Yadava K , et al. Gut microbiota metabolism of dietary fiber influences allergic airway disease and hematopoiesis. Nat Med. 2014;20:159‐166. 24390308 10.1038/nm.3444

[21] Beeler‐Marfisi J , Clark ME , Wen X , et al. Experimental induction of recurrent airway obstruction with inhaled fungal spores, lipopolysaccharide, and silica microspheres in horses. Am J Vet Res. 2010;71:682‐689. 20513185 10.2460/ajvr.71.6.682

[22] Pirie RS , Collie DD , Dixon PM , McGorum BC . Inhaled endotoxin and organic dust particulates have synergistic proinflammatory effects in equine heaves (organic dust‐induced asthma). Clin Exp Allergy. 2003;33:676‐683. 12752598 10.1046/j.1365-2222.2003.01640.x

[23] Wang Q , Li F , Liang B , et al. A metagenome‐wide association study of gut microbiota in asthma in UK adults. BMC Microbiol. 2018;18:114. 30208875 10.1186/s12866-018-1257-x PMC6134768

[24] Salem SE , Maddox TW , Berg A , et al. Variation in faecal microbiota in a group of horses managed at pasture over a 12‐month period. Sci Rep. 2018;8:8510. 29855517 10.1038/s41598-018-26930-3 PMC5981443

[25] Fernandes KA , Kittelmann S , Rogers CW , et al. Faecal microbiota of forage‐fed horses in New Zealand and the population dynamics of microbial communities following dietary change. PLoS One. 2014;9:e112846. 25383707 10.1371/journal.pone.0112846 PMC4226576

[26] David LA , Maurice CF , Carmody RN , et al. Diet rapidly and reproducibly alters the human gut microbiome. Nature. 2014;505:559‐563. 24336217 10.1038/nature12820 PMC3957428

[27] Costa MC , Stampfli HR , Arroyo LG , et al. Changes in the equine fecal microbiota associated with the use of systemic antimicrobial drugs. BMC Vet Res. 2015;11:19. 25644524 10.1186/s12917-015-0335-7 PMC4323147

[28] Costa MC , Stampfli HR , Allen‐Vercoe E , Weese JS . Development of the faecal microbiota in foals. Equine Vet J. 2016;48:681‐688. 26518456 10.1111/evj.12532

[29] Dougal K , de la Fuente G , Harris PA , Girdwood SE , Pinloche E , Newbold CJ . Identification of a core bacterial community within the large intestine of the horse. PLoS One. 2013;8:e77660. 24204908 10.1371/journal.pone.0077660 PMC3812009

[30] Steelman SM , Chowdhary BP , Dowd S , Suchodolski J , Janečka JE . Pyrosequencing of 16S rRNA genes in fecal samples reveals high diversity of hindgut microflora in horses and potential links to chronic laminitis. BMC Vet Res. 2012;8:231. 23186268 10.1186/1746-6148-8-231 PMC3538718

[31] Jochmans‐Lemoine A , Picotte K , Beauchamp G , Vargas A , Lavoie JP . Effects of a propriety oiled mixed hay feeding system on lung function, neutrophilic airway inflammation and oxidative stress in severe asthmatic horses. Equine Vet J. 2019;1‐8. 10.1111/evj.13218 31802526

